# The Gut‐Brain Axis in Parkinson disease: Emerging Concepts and Therapeutic Implications

**DOI:** 10.1002/mdc3.70029

**Published:** 2025-03-13

**Authors:** Elisa Menozzi, Anthony H.V. Schapira, Per Borghammer

**Affiliations:** ^1^ Department of Clinical and Movement Neurosciences UCL Queen Square Institute of Neurology London UK; ^2^ Aligning Science Across Parkinson's (ASAP) Collaborative Research Network Chevy Chase Maryland USA; ^3^ Department of Clinical Medicine Aarhus University Aarhus Denmark; ^4^ Department of Nuclear Medicine and PET Aarhus University Hospital Aarhus Denmark

**Keywords:** enteroendocrine cells, gut‐brain axis, microbiome, Parkinson disease

## Abstract

**Background:**

The gut‐brain axis, i.e. the bidirectional communication system between the gut and the brain, has become of central importance in Parkinson disease (PD) research over the past 20 years.

**Aims:**

We aimed to describe the milestones of the gut‐brain axis research in PD and the development of theories proposing the involvement of the gastrointestinal tract in PD pathogenesis.

**Methods:**

We searched PubMed using the terms ‘gut‐brain axis’ AND ‘Parkinson disease’, and selected relevant articles to provide the foundation for reconstructing an historical overview of the gut‐brain axis research in PD.

**Results:**

Mounting evidence from preclinical, clinical and post‐mortem studies suggests that a subgroup of PD patients present with a range of prodromal symptoms (e.g., autonomic dysfunction, rapid eye movement sleep behaviour disorder) which reflect initial accumulation and later spread of pathological α‐synuclein rostrally from the gastrointestinal tract (“body‐first” PD). Through neural connections along the gut‐brain axis, pathological α‐synuclein may spread to the brain, producing clinically manifest disease. Recently, two mechanisms involving the gut‐brain axis have attracted increasing attention for their role in PD pathogenesis and progression, namely the perturbation of the composition of the microorganisms living in the gut (the gut microbiome), and the dysfunction of enteroendocrine cells.

**Conclusion:**

Treatments targeting the gut‐brain axis, especially the gut microbiome and the enteroendocrine cells pathway, could potentially slow disease progression or even prevent disease onset. Among these, pre/probiotics, faecal microbiota transplantation, and glucagon‐like peptide‐1 receptor agonists, have entered advanced stages of clinical trials in humans and shown potential symptomatic and disease‐modifying effects.

## The Gut‐Brain Axis: General Concepts

The bidirectional communication between the brain and the gut represents a growing and exciting area of research which has its roots in the eighteenth century, when the idea that digestion, emotions and identity were linked in a bidirectional fashion was first proposed.^1^ The concept of “nervous sympathy”, introduced by the Scottish physician Robert Whytt in 1765, defined the communication network by which all internal body organs, including the brain and gut, were connected.^2^ In the nineteenth century, the anatomy of the gut‐brain axis was outlined.^1^ During the late twentieth and early twenty‐first century a third player in this axis, the gut microbiome (ie, the trillions of microbes living within the gut) came to light,^3^, ^4^, ^5^, ^6^, ^7^ alongside evidence supporting alterations of the microbiome‐gut‐brain axis in several neurodevelopmental, psychiatric and neurological disorders.^3^ Among the latter, Parkinson disease (PD) has been the most studied.^3^

**Figure 1:**
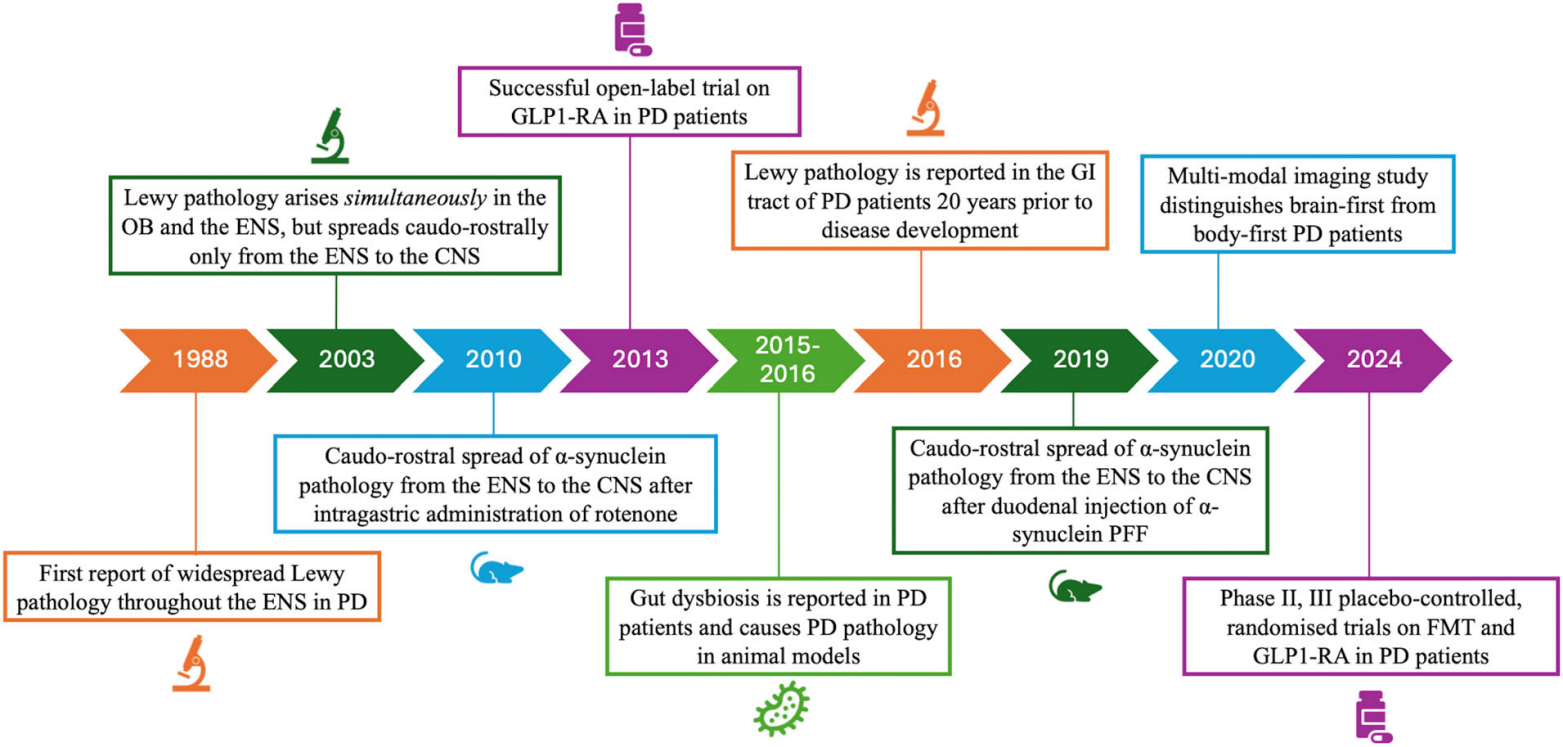
Historical milestones of the gut‐brain axis research in PD. CNS, central nervous system; ENS, enteric nervous system; FMT, faecal microbiota transplantation; GLP‐1 RA, glucagon‐like‐1 receptor agonists; OB, olfactory bulb; PD, Parkinson disease.

This review presents a historical overview of the milestones of the gut‐brain axis research in PD and the evolution of the theories supporting a role of the gastrointestinal tract in the pathogenesis of this condition (Fig. 1), with a particular focus on those pathways which may be potential therapeutic targets in PD, including the gut microbiome and enteroendocrine system.

## Methods

PubMed was searched on June 1, 2024, for articles published in English using the terms “gut‐brain axis” AND “Parkinson disease” in the Title/Abstract. Abstracts were scrutinized and relevant articles were selected based on content and impact. Additional studies outside this time window which were considered of relevance were also included.

## The Origins and Evolution of Gut‐Brain Axis Theories in Parkinson disease

Gastrointestinal disorders, including constipation, dysphagia, nausea and vomiting, represent some of the major sources of disability and reduced quality of life for PD patients.^8^, ^9^ In some cases, constipation can emerge more than 20 years before motor manifestations.^10^, ^11^

It was observed in 1928 that Lewy pathology, that is the aggregation of α‐synuclein into inclusion bodies,^12^ involves not only the central nervous system (CNS) but also the peripheral sympathetic nervous system in PD.^13^ Lewy pathology within the enteric nervous system (ENS) was then reported in 1980's.^14^, ^15^ Nevertheless, the importance of these findings was not appreciated until 2003, when Heiko Braak proposed that PD “might originate outside of the CNS, caused by a yet unidentified pathogen that is capable of passing the mucosal barrier of the gastrointestinal tract and, via postganglionic enteric neurons, enter the CNS”.^16^ Since then, an increasing body of evidence supports that the gastrointestinal tract plays a role in the pathogenesis and progression of PD.

### Gastrointestinal α‐Synuclein Accumulation

After the initial reports from the 1980's,^14^, ^15^ several postmortem studies have unequivocally demonstrated that aggregated and phosphorylated α‐synuclein deposits are present throughout the myenteric and submucosal plexus of patients with PD or dementia with Lewy bodies (DLB),^17^, ^18^, ^19^, ^20^ and can be detected up to 20 years before diagnosis.^21^ The density of α‐synuclein deposits mirrors the vagal innervation, being most severe in the lower esophagus and least in the colon.^15^, ^18^, ^19^

Several studies of living patients have confirmed these postmortem results, reporting the presence of aggregated α‐synuclein in biopsies throughout the gastrointestinal tract from both patients with manifest PD and prodromal patients with isolated rapid eye movement (REM) sleep behavior disorder (iRBD),^21^, ^22^, ^23^, ^24^, ^25^, ^26^ a condition associated with >80% probability of conversion to PD.^27^ Imaging studies from iRBD cohorts also showed significantly increased gastrointestinal transit time and colonic volume, which deteriorated over time.^28^, ^29^ These data, alongside the evidence that PD patients with REM sleep behavior disorder (RBD) seemed to show higher rates of enteric phosphorylated α‐synuclein positivity compared to non‐RBD PD patients,^30^ suggest that iRBD patients are characterized by progressive gastrointestinal dysfunction that precedes brain dysfunction, and it seems probable that this dysfunction relates to the progressive and widespread accumulation of pathological α‐synuclein in the autonomic nervous system.

In a few studies, PD patients did not display more phosphorylated α‐synuclein aggregates than control subjects.^31^, ^32^ These discrepant results might be due to differences in study design, for example localization of biopsies, heterogeneity in the techniques applied, and disease stage at which the samples were taken. Notably, the myenteric plexus displayed the highest rate of positivity, whereas the mucosa and submucosa layers were less frequently positive.^20^ Overall, this means that small submucosal colon biopsies probably have the lowest sensitivity, whereas deeper gastro‐esophageal biopsies including the myenteric plexus show the highest sensitivity.

### Retrograde Spread of α‐Synuclein from the Gastrointestinal Tract to the Brain Via the Vagus Nerve

Following these initial findings, and given that α‐synuclein can spread from cell to cell,^33^ numerous preclinical studies supported a role of gut‐seeded α‐synuclein pathology in PD pathogenesis, by showing that α‐synuclein could propagate from the gut to the brain.

The principal route connecting gut‐to‐brain is the vagus nerve. Epidemiological studies suggested a protective role of vagotomy, especially truncal vagotomy, towards PD development,^34^, ^35^ but the strongest evidence that the vagus could act as route of transmission for α‐synuclein spread came from animal models. Intragastric administration of low doses of rotenone, a pesticide that reproduces PD pathological changes by inhibiting complex I of the mitochondrial respiratory chain, increased phosphorylated α‐synuclein in the ENS, dorsal motor nucleus of the vagus (DMV) and substantia nigra pars compacta (SNpc), in a sequential, time‐dependent fashion, and only in synaptically connected neuronal structures.^36^ Hemivagotomy and partial sympathectomy prevented the spread of PD pathology within these connected structures.^37^ Other models achieved the same results by injecting various forms of α‐synuclein (recombinant α‐synuclein, pre‐formed fibrils‐PFF) in the gut.^38^, ^39^, ^40^, ^41^ However, some of these models reported that standalone gut‐seeded α‐synuclein was not sufficient to promote spreading of α‐synuclein to the brain, and that time might play a pivotal role in this process. In one model using gastric PFF injection, Lewy pathology was detected in the DMV but not in more rostral structures, after 12 months post‐inoculation.^39^ When PFFs were injected in the duodenum, old mice (16 months‐old) showed progression of phosphorylated α‐synuclein to the brain and decreased striatal dopamine innervation, whereas young mice (8‐10 week‐old) developed gastrointestinal alterations and accumulation of phosphorylated α‐synuclein only in myenteric neurons.^41^ Another recent model using injection of α‐synuclein PFF into the muscularis layers of the duodenum and stomach demonstrated the retrograde propagation of phosphorylated α‐synuclein to the upper gastrointestinal tract, heart, and CNS, and anterograde propagation to the colon via the vagus. The time‐dependent propagation of phosphorylated α‐synuclein in the CNS correlated with the development of non‐motor symptoms, for example impaired gastrointestinal motility (DMV) at 2 weeks post‐injection, hyposmia (parabigeminal nucleus and dorsal raphe) after 1 month, sleep disturbances (pons and midbrain) after 3 months, and cognitive disturbances (striatum and frontal cortex) at 6–7 months post‐injection.^42^ Bidirectional propagation of α‐synuclein via the vagus (duodenum‐to‐DMV‐to‐stomach) and propagation of α‐synuclein to the intermediolateral nucleus of the spinal cord (IML) and the heart, was also shown after duodenal PFF injection in transgenic rats with excessive levels of human α‐synuclein.^43^ Some studies did not reproduce these findings in rats or non‐human primates, failing to demonstrate permanent α‐synuclein pathology in the CNS after enteric injections of PFF or adeno‐associated virus overexpressing the A53T mutant α‐synuclein.^44^ These discrepancies might be secondary to differences in sites of injection, age and sex of animals.

The proposed anatomical route of the bidirectional propagation of α‐synuclein from the GI tract to both parasympathetic and sympathetic structures involves spreading via coeliac and mesenteric ganglia and then to the sympathetic trunk, including stellate ganglion, heart, and the IML of the spinal cord.^43^ Human postmortem studies confirmed the presence of Lewy pathology in all above‐mentioned structures.^19^, ^20^, ^45^, ^46^ Considering that a number of cases displayed isolated Lewy pathology in the sympathetic trunk and heart without any pathology in the CNS,^20^, ^46^ it can be hypothesized that in some cases the earliest detectable Lewy pathology begins outside the CNS, most likely in the gut, and sometimes with predominant spreading via sympathetic connections preceding vagal propagation.

### The Brain‐First and Body‐First Model

Converging evidence from postmortem and animal models supports the existence of a PD subtype, characterized by initial development of α‐synuclein pathology in the ENS, with subsequent propagation to the DMV, sympathetic system, brainstem, and the rest of the CNS (body‐first).^47^, ^48^, ^49^, ^50^ However, postmortem studies showed than a number of PD cases display a limbic‐predominant distribution of α‐synuclein inclusions with less pathology in the brainstem.^51^, ^52^ This suggests the existence of another PD subtype, in which α‐synuclein pathology may originate in the olfactory bulb (OB) and/or limbic system (brain‐first), and subsequently descend to the brainstem and peripheral nervous system.^48^ This body‐first/brain‐first model was supported by imaging and clinical studies, evaluating cases with prodromal iRBD or with early‐stage PD with or without premotor RBD. Individuals with PD and premotor RBD showed more severe cardiac sympathetic denervation, colon parasympathetic denervation, delayed colon transit time and enlarged colonic volume when compared with PD subjects without RBD, despite comparable levels of dopaminergic degeneration.^48^ Individuals with iRBD and body‐first PD both tended to exhibit more symmetric putaminal dopaminergic degeneration when compared with brain‐first PD.^53^ Of note, pure autonomic failure (PAF) may represent a subtype of body‐first Lewy body disease characterized by particularly early and severe sympathetic dysfunction.^54^ Such Lewy‐body positive PAF patients almost universally display severe cardiac sympathetic denervation,^55^, ^56^, ^57^ and may convert to PD or DLB upon follow‐up.^58^ Interestingly, PAF patients who present with mild degree of cardiovagal impairment, but severe urinary dysfunction and sweat abnormalities, tend to phenoconvert to multiple system atrophy (MSA) in longitudinal studies.^58^, ^59^

Aiming to explain not only the origin of PD and DLB but also the heterogeneity observed in the prodromal phase and clinical progression of these patients, the body‐first/brain‐first model was further expanded into the *α‐synuclein, origin and connectome* (SOC) model. This model proposes that α‐synuclein pathology, (a) originates in one single location in most individuals (either OB/amygdala or autonomic/ENS), and (b) propagates following the neural connectome.^60^ According to the model, body‐first patients have a longer prodromal phase characterized by the development of RBD and dysautonomia, and present with a greater burden of α‐synuclein pathology at the time of motor symptoms development. Relatively symmetrical propagation of α‐synuclein pathology from the ENS through both the right and left vagus to the CNS leads to more symmetric parkinsonism, accelerated cognitive decline and more severe non‐motor symptoms.^60^ In contrast, brain‐first patients have a shorter prodromal phase and initially display more asymmetric parkinsonism, since pathological α‐synuclein, originating in one OB, propagates to the ipsilateral amygdala and SNpc through the highly lateralized brain connectome. Brain‐first patients typically do not develop RBD or autonomic non‐motor symptoms during the prodromal stage, but most will eventually develop these symptoms after diagnosis as α‐synuclein pathology spreads in a rostro‐caudal direction.^60^ Clinical data strongly support that body‐first Lewy body disease is a more “malignant phenotype” with faster progression and development of dementia. For instance, DLB patients exhibit more constipation, urinary dysfunction and orthostatic hypotension (ie, a stronger body‐first profile) 5–10 years before diagnosis compared to PD.^61^ In a large prospective multi‐center study of iRBD patients, approximately 45% of cases converted directly to DLB, and not PD.^62^ Furthermore, early development of autonomic dysfunction has been consistently associated with faster progression and shorter survival.^63^, ^64^ The hypothesis behind a faster progression to dementia in body‐first PD subtype is that at the time of diagnosis (ie, onset of motor symptoms), body‐first PD patients present with a higher burden of α‐synuclein pathology of all important modulatory neurotransmitter nuclei in the brainstem and basal forebrain, including the raphe nuclei, locus coeruleus, substantia nigra, and nucleus basalis of Meynert, compared to brain‐first.^60^ Considering that the involvement of these brainstem modulatory nuclei parallels the formation of widespread cortical Lewy pathology,^60^ a key neuropathological substrate of dementia in Lewy body disease,^65^ the cognitive decline in body‐first PD patients might be accelerated.

A growing number of studies have tested the predictions of the SOC model (recently reviewed^47^). Notably, one study used data from 919 PD patients and 88 iRBD patients from three independent cohorts: PD patients with RBD showed earlier disease onset and developed earlier changes in non‐motor symptoms, especially decline and dysautonomia, compared with PD patients without RBD.^66^ In another study using similar datasets, PD individuals defined as fast‐progressing (body‐first) showed a higher frequency of RBD symptoms, more severe non‐motor symptoms and cognitive decline, and more symmetrical dopaminergic loss at baseline compared to slow‐progressing (brain‐first).^67^ Thus, most studies report findings in support of the model with a few exceptions,^68^ and further validation based on advanced disease modeling techniques is warranted.

## The Gut Microbiome: An Additional Player in the Axis

Different origins and spreading routes of pathological α‐synuclein provide a plausible biological explanation for the observed heterogeneity of PD, but do not explain how gut pathology in PD patients might develop in the first place. In the last decade, changes in the gut microbiome have generated the greatest attention as possible contributors to PD pathogenesis.^3^ Preclinical evidence supports a pathogenic role of the gut microbiome in PD, whereas results from human studies do not fully clarify whether alterations of the gut microbiome are primary contributors to pathogenesis or a mere consequence to the disease or its treatments.^69^

### Gut Dysbiosis in Animal Models: A Possible Catalyst of PD Pathogenesis

The first evidence suggesting a possible causative role played by the gut microbiome in PD pathogenesis dates back to 2016, when a seminal study showed that human α‐synuclein overexpressing (ASO) mice kept under germ‐free conditions, or after bacteria depletion with antibiotics, displayed fewer α‐synuclein inclusions, reduced microglial activation, and attenuated motor deficits compared to animals harboring a complex microbiome.^70^ When ASO mice received fecal microbiota transplantation (FMT) from people with PD, they showed more severe motor impairment compared to genotype‐matched mice receiving FMT from healthy controls.^70^ Subsequently, using a different PD animal model, it was shown that FMT from animals treated with 1‐methyl‐4‐phenyl‐1,2,3,6‐tetrahydropyridine (MPTP) by intraperitoneal injection promoted motor dysfunction alongside reduction of striatal dopamine and serotonin levels in normal mice.^71^ Similarly, FMT from healthy mice showed amelioration of motor and gastrointestinal abnormalities, neuroinflammation and striatal dopaminergic content in several animal models.^71^, ^72^

### Gut Dysbiosis in Manifest and Prodromal PD Individuals: Cause or Consequence?

After the first case–control study in 2015 reporting disruption of the gut microbiome homeostasis in individuals with PD,^73^ many other studies and three meta‐analyses have been published, showing several families, genera and bacterial species being differentially abundant in PD compared to controls.^74^, ^75^, ^76^, ^77^, ^78^, ^79^

Among the various findings, one of the most consistent is the relative decreased abundance of bacteria involved in the production of short‐chain fatty acids (SCFAs) such as species belonging to genera *Roseburia*, *Faecalibacterium* and *Prevotella*.^74^, ^75^, ^76^, ^77^, ^79^ This is mirrored by the detection of reduced levels of SCFAs (mainly, acetate, propionate and butyrate) in fecal samples of PD patients.^80^, ^81^, ^82^, ^83^, ^84^ SCFAs are essential microbiota‐derived metabolites, produced by the anaerobic fermentation of dietary fibre^85^; they represent the preferential energy source for colonocytes and promote intestinal barrier integrity.^3^, ^86^, ^87^ Hence, SCFA depletion can increase intestinal inflammation and barrier permeability (“leaky gut”), both well‐established features in PD patients.^88^, ^89^, ^90^ Such gut microbiome perturbations, involving a shift towards a proinflammatory state through depletion of gut‐microbial anti‐inflammatory activity, have been confirmed by evaluation of microbiome functions.^78^, ^91^ That given, it could be hypothesized that alterations in SCFA‐producing bacteria might play a role in PD pathogenesis, however preliminary data on SCFA‐producing bacteria abundance in at‐risk populations such as iRBD are contrasting,^92^, ^93^ so further confirmation is needed.

Another consistent finding reported across PD microbiome studies is the enrichment of genera such as *Lactobacillus* and *Bifidobacterium*.^74^ Interestingly, enrichment of certain *Bifidobacterium* species was also detected in constipated individuals or individuals with constipation combined with RBD and hyposmia,^94^ suggesting a potential role in primary pathogenesis. Nevertheless, the role of these microbial species as contributors to PD pathogenesis remains controversial. The genera *Bifidobacterium* and *Lactobacillus* are generally associated with beneficial effects and thus included in many probiotics’ formulations.^77^ Enrichment of *Bifidobacterium* species was not detected in early, levodopa naïve PD patients,^78^ nor in iRBD individuals.^92^ Moreover, *Bifidobacterium* abundance was associated with higher levodopa dosage in treated PD patients, and *in vitro* studies showed that certain species belonging to this genus, such as *B. bifidum* and *B. longum*, were able to metabolize levodopa,^95^ suggesting that changes in *Bifidobacterium* abundance might be a consequence of the disease or levodopa treatment, rather than a primary mechanism of disease development.

The increased abundance of genus *Akkermansia*, in particular *A. muciniphila*, in PD has also been consistently reported in many studies,^75^, ^76^ although not confirmed in a recent large metagenomics study.^79^ Genus *Akkermansia* was also enriched in several cohorts of iRBD.^92^, ^93^ *A. muciniphila* plays a critical role in the maintenance of intestinal mucus integrity and dynamics, thus protecting the host from LPS‐induced endotoxemia.^96^ Its abundance is generally correlated with human health and found to be significantly reduced in multiple diseases,^97^ so the findings in PD seem surprising. It has been hypothesized that the observed enrichment in *Akkermansia* in PD is a compensatory effect in response to the under‐representation of anti‐inflammatory and mucin‐degrader species (genus *Prevotella* or *Ruminococcus*), aiming to maintain the mucin degradation status.^98^ Proteins obtained from *A. muciniphila*‐conditioned medium induce mitochondrial calcium overload and phosphorylation and aggregation of α‐synuclein in enteroendocrine cells.^99^ Whether *Akkermansia* abundance plays a role in PD pathogenesis remains, though, elusive.

### Possible Contributors to Gut Microbiome Changes in PD

In support of a possible primary pathogenic role of gut dysbiosis in PD, there is some preclinical evidence that either the exposure to certain environmental factors (eg, organochlorine pesticides and dry‐cleaning chemicals) or the presence of gastrointestinal conditions (eg, inflammatory bowel diseases—IBD) which have been reported to have an association with an increased risk of PD,^100^, ^101^ can induce gut microbiome changes similar to those observed in PD and presented above. For instance, depletion in SCFA‐producing bacteria and increase in *Bifidobacteria*, were detected in adult rats treated with trichloroethylene (TCE) via oral gavage.^102^ Exposure to pesticides such as paraquat increased the abundance of *Lactobacillaceae* and *Akkermansiaceae*, and decreased that of anti‐inflammatory *Lachnospiraceae*, *Ruminococcaceae* and *Prevotellaceae* in adult mice, although the doses used were probably not comparable to human exposure.^103^ Similarly, reduced levels of SCFA‐producing bacteria was shown across PD and IBD patients.^104^

We acknowledge that many other factors including aging, genetic risk factors, etc, need to be considered in the pathogenesis of PD. However, the comparison of the PD gut microbiome with the gut microbiomes of other conditions associated with increased risk of PD can shed light on distinctive microbiome signatures which might help understand the pathogenic processes underlying PD.

### Impact of Gut Dysbiosis on Clinical Phenotype and Progression

Beyond the potential pathogenic role of gut microbiome in PD, there is some preliminary evidence suggesting that the gut microbiome composition might impact on clinical phenotypes and progression. A reduction in total bacterial count and specific species, including *Prevotella*, was observed over a 2‐year follow‐up study,^105^ with lower levels of *Bifidobacterium* at baseline being associated with faster progression and worsening of hallucinations/delusions.^105^ In another study with mean follow‐up of 2.25 years, no significant changes in gut microbiome structure were observed over time, however, the levels of genus *Prevotella* were found to be higher in stable PD patients compared with faster progressing at both baseline and follow‐up, supporting a protective role for this genus.^106^ These findings highlight the potential use of microbiome targeted therapies as disease‐modifying strategies. Additional longitudinal studies with longer follow‐up times are needed to detect meaningful clinical differences and prove their possible associations with microbiome features.

## The Enteroendocrine System: An Alternative Gut‐Brain Connection

The potential relevance of another gut‐brain connection, which includes gut enteroendocrine cells (EECs), vagal afferents and the brainstem, has recently emerged in PD research.^107^, ^108^, ^109^, ^110^, ^111^

EECs are specialized intestinal chemosensory cells, able to transduce luminal signals, including microbial components and metabolites (eg, lipopolysaccharide‐LPS, SCFA), nutrients (eg, sugar, amino acids, free fatty acids), and noxious stimuli, through specialized receptors.^112^ Representing 1% of the total gut epithelial cells and mainly located in the small intestine and colon,^113^ EECs attracted the interest of the PD field as they not only display neuronal‐like properties including synaptic features and electrical excitability,^108^, ^114^ but they also express α‐synuclein.^115^ Since intracellular levels of α‐synuclein protein were increased in EECs upon exposure to bacterial components such as LPS or lipopeptide, it has been proposed that EECs could be the initial site of PD pathology in the gut under specific microbial circumstances.^116^ How α‐synuclein would then spread from EECs remains elusive, especially considering that myelinated viscerosensory fibers are less prone to develop and transport α‐synuclein pathology compared to poorly myelinated visceromotor fibers.^16^

The function of EECs has been reported to be impaired in PD. Postprandial systemic levels of glucagon‐like peptide‐1 (GLP‐1), secreted by a subtype of EECs (L‐cells) in response to a meal,^114^ were found to be reduced in patients with PD compared to household controls consuming the same meal.^117^ Regarding the mechanisms underlying the attenuated GLP‐1 responses observed in PD, it has been suggested this is linked to SCFA metabolism. In fact, SCFAs can promote GLP‐1 secretion by binding to the transmembrane free fatty acid receptor 2 (FFAR2) expressed on L‐cells,^118^, ^119^ and prebiotic supplementation aimed at enhancing SCFA levels was able to increase GLP‐1 levels in plasma in a mouse model.^119^ GLP‐1 is a potent anorexigenic hormone which regulates glucose homeostasis by increasing pancreatic insulin secretion, suppressing glucagon secretion, and slowing gastric emptying.^120^ GLP‐1 receptors are expressed in gut enteric neurons, vagal afferents, and CNS.^3^ Beyond its glucose‐lowering effect, activation of the GLP‐1 pathway can induce a wide range of neuroprotective and anti‐inflammatory effects (eg, reduction of oxidative stress, inhibition of the NF‐κB pathway, and influence on mitochondrial function).^121^, ^122^, ^123^ Beneficial effects of enhanced GLP‐1 signaling were shown in several PD animal models after treatment with GLP‐1 receptor agonists (GLP‐1RA), drugs currently approved in the treatment of type 2 diabetes (eg, exenatide, a synthetic form of exendin‐4, liraglutide, semaglutide, lixisenatide).^86^ For instance, in MPTP mouse models, treatment with exendin‐4, a long‐acting GLP‐1RA, prevented dopaminergic neurons degeneration and motor impairment, preserved dopamine levels,^124^ and reduced microglia activation and inflammation.^125^ Liraglutide, together with another anti‐diabetic drug sitagliptin, improved motor performances, rescued neuronal loss, increased dopamine levels and reduced inflammation in a rotenone model.^126^ These promising preclinical results have led to testing these drugs in clinical trials (see below).^127^, ^128^, ^129^

In conclusion, although the pathogenic role of EECs in initiating enteric α‐synuclein pathology and the pathophysiological role of altered GLP‐1 responses in PD remain controversial, the dysfunction of the enteroendocrine system, possibly via alterations of the gut microbiome, represents an interesting therapeutic target in PD.

## Novel Targeted Treatments Within the Gut‐Brain Axis in PD

The considerable advances in the understanding of the gut‐brain axis in PD have led to several novel potential therapeutic strategies, mainly targeting the gut microbiome and GLP‐1 pathway. Some of these treatments have already reached advanced clinical trial testing, including randomized‐controlled trials (RCT).

In this section, we will briefly review recent evidence from clinical trials, mainly focusing on phase II‐III RCTs when available. More comprehensive reviews of the preclinical evidence underlying these therapeutic strategies have been published recently.^123^, ^130^

### Dietary Interventions

The mediterranean diet (MedDiet) is the most intensely studied dietary intervention in PD. High adherence to MedDiet reduces the risk of PD development,^131^, ^132^, ^133^ with one unit increase in the adherence score to MedDiet being associated with a 29% lower risk of PD in women above 65 years of age.^133^ The protective effects of MedDiet are likely linked to both its anti‐oxidative and anti‐inflammatory properties due to intake of polyphenols, polyunsaturated fatty acids, dietary fibers, and antioxidants found in whole grains, fruits and vegetables, nuts and red wine,^134^ and its effects on gut microbiome composition, including increased levels of anti‐inflammatory SCFA‐producing bacteria.^135^ In two RCTs, high adherence to MedDiet for 10 weeks improved cognitive functions and favored antioxidant effects in PD patients compared to habitual diet.^136^, ^137^ Results from an 8‐week RCT investigating the effect of MedDiet on constipation are awaited soon (MEDI‐PD; ClinicalTrials.gov ID: NCT04683900).^138^

One RCT assigned PD patients to follow either a low‐fat or ketogenic diet for 8 weeks and reported improvements in disease severity in both groups as measured by the total Movement Disorders Society‐Unified PD Rating Scale (MDS‐UPDRS) score.^139^ The ketogenic diet showed superiority over the low‐fat diet in decreasing non‐motor symptoms severity as shown by more significant reduction in MDS‐UPDRS part I score.^139^ Another proof‐of‐concept, cross‐over study in participants with PD receiving an 8‐week Mediterranean ketogenic diet vs Mediterranean diet supplemented with medium‐chain triglycerides, separated by an 8‐week washout period, is currently underway with completion expected in 2025 (ClinicalTrials.gov ID: NCT05469997). Changes in gut inflammation markers (fecal and serum calprotectin), gut microbiome composition and SCFA levels are among the primary outcomes analyzed.

### Probiotics and Prebiotics

Alternative approaches to target the microbiome‐gut‐brain axis include probiotics (“live microorganisms which when administered in adequate amounts confer a health benefit on the host”) and prebiotics (“substrates that are selectively used by host microorganisms conferring a health benefit”).^3^, ^140^, ^141^, ^142^ The available evidence prompted the MDS Evidence Based Medicine Committee to classify the use of probiotics and prebiotic fibers as efficacious, safe and clinically useful in clinical practice.^143^ However, there is no general consensus on the use of such treatments yet.

Results from nine RCTs testing different combinations of probiotics (mainly strains of genera *Lactobacillus* and *Bifidobacterium*) showed a consistent beneficial effect on constipation.^144^, ^145^, ^146^, ^147^, ^148^, ^149^, ^150^, ^151^, ^152^ A recent meta‐analysis confirmed that probiotic treatments significantly improved motor symptoms, anxiety and depression, alleviated constipation, and reduced the use of laxatives in PD patients.^153^ These studies all differed in terms of supplemented species or strain types, duration of intervention, and dose of probiotics,^153^ thus limiting smooth translation of these results into clinical practice.

Current evidence for prebiotics is limited to open‐label studies, with reported post‐intervention amelioration in mood, gastrointestinal and motor functions, and a positive safety and tolerability profile.^154^, ^155^, ^156^, ^157^ Modifications of gut microbiome composition/functionality (ie, increased abundance of SCFA‐producing bacteria, reduced abundance of putative pro‐inflammatory bacteria, and increased SCFA levels), and reduced levels of intestinal inflammation markers such as calprotectin, have been reported post‐intervention.^154^, ^155^ The effect of a 30‐day supplementation with tributyrin, a butyrate prodrug which delivers butyric acid directly into the gastrointestinal tract, was evaluated in two healthy controls and eight PD patients. A trend towards improvement in dopamine‐refractory symptoms such as postural instability and gait difficulties was observed post‐intervention, and this was associated with increased availability of brain butyrate in several brain regions as measured by ^11^C‐butyrate PET imaging.^158^ The non‐blinded design and small sample sizes of the studies were significant limitations.

### Fecal Microbiota Transplantation

FMT, that is infusion of filtered fecal material from a donor to a host with the goal of replacing the recipient's gut microbiome, is a very effective treatment for recurrent *Clostridium difficile* infection.^159^

Several case reports, case series and open‐label studies conducted in PD cohorts demonstrated that FMT was safe, relatively well‐tolerated, and caused subjective and objective improvement in motor and non‐motor symptoms.^160^, ^161^, ^162^ These promising preliminary data have prompted testing of FMT in five placebo‐controlled, RCTs, with results available for four of them (summarized in Table 1).^163^, ^164^, ^165^, ^166^ Three of four studies evaluated changes in global or motor function as primary outcomes. Two studies reported a significant improvement in the active arm either at 12 weeks or 12 months after the intervention,^164^, ^165^ whereas the third study reported no changes in motor function between the two arms at 6 months post‐FMT, although a greater increase in medication dose was observed in the placebo arm.^166^ Microbiome changes after FMT were explored only in three studies. Two studies reported beneficial changes in the donor‐FMT arm, with a shift towards anti‐inflammatory microbial species, possibly reflecting the positive clinical outcomes.^163^, ^164^ In the study reporting negative clinical results, the dysbiosis status changed to non‐dysbiotic after donor‐FMT in only 3% of individuals at 12 months versus 33% in the placebo arm.^166^

**TABLE 1:** Summary of placebo‐control, RCTs on FMT conducted in PD populations

Type of intervention	Duration of intervention	Clinical trials ID	Study population	Sample size (treatment vs. placebo)	Primary outcome	Safety	Other observations	Ref.
Capsules containing freeze‐dried material from donors (50 g of donor feces) or placebo	Once a week for 3 consecutive weeks	NA	Mild to moderate PD (H&Y 1–3)	27 vs. 27	Greater improvement of MDS‐UPDRS total score at 12 weeks in FMT group	No SAE. Minor GI disturbances. Improvement in GI function (increased stool frequency) in FMT group	‐	164
Oral dose of lyophilized product (60 g of donor feces) or placebo	Twice a week for 12 weeks	NCT03671785	Mild to moderate PD (H&Y 1–2.5), with constipation	7 vs. 4	Safe treatment, only mild–moderate upper GI disturbances	‐	No clinical comparison as groups differed at baseline (worse MDS‐UPDRS total score in placebo group)	163
Single‐dose nasojejunal administration of FMT from healthy donor (50 g donor of feces) or own feces (placebo group)	Once	NCT03808389	Moderate PD (H& Y 2 in OFF state)	22 vs. 24	Greater improvement of MDS‐UPDRS part III in OFF in FMT group at 12 months	No SAE. Mild GI disturbances after first week post‐FMT, more frequent in donor‐FMT arm (59%) than placebo‐FMT arm (25%)	No effect on MDS‐UPDRS part I, II, IV, LEDD, cognition or mood	165
Single‐dose FMT via colonoscopy from healthy donors (30 g of feces) or placebo (saline and glycerol)	Once	NCT04854291	Mild to moderate PD (H& Y 2)	30 vs. 15	No effect on MDS‐UPDRS part I‐III at 6 months in FMT group	3 SAE in FMT group but unrelated to the intervention. Greater frequency of GI disturbances in FMT arm (53%) than placebo arm (7%)	Increased LEDD in placebo arm	166 ^a^

Abbreviations: FMT, fecal microbiota transplantation; GI, gastrointestinal; H&Y, Hoehn & Yahr; LEDD, levodopa equivalent daily dose; MDS‐UPDRS, Movement Disorders Society‐Unified Parkinson Disease Rating Scale; SAE, serious adverse events.

^a^ This study has been published outside the time window for our literature search but was nevertheless included.

These contrasting results raise some concerns about the design of future FMT studies in PD. Consensus on technical aspects including choice of donors, types of placebo (auto‐transplant or inert placebo), preparation and administration routes of the fecal material, and selection of recipients, should be addressed before launching new trials.^166^ For instance, baseline gut microbiome features and gastrointestinal function of recipients might be important to distinguish responsive from unresponsive individuals and thus increase the probability of positive results. In the meantime, another single‐center, pilot study evaluating FMT in advanced PD (>5 years disease duration and presence of motor complications) has already started rectruitment.^167^

### GLP‐1RA

The GLP‐1 pathway constitutes another exciting therapeutic target along the gut‐brain axis. Evidence from phase II and III RCTs is already available for several GLP‐1RA.

Exenatide was found to improve motor outcome after 48 weeks, which persisted after a 12‐week washout period, although to a lesser extent, in a phase II RCT.^127^ Adverse events, including weight loss and gastrointestinal symptoms, occurred at similar frequencies in treated and placebo arms.^127^ Even though these results suggested a disease‐modifying effect, it is possible that the observed benefits were secondary to long‐lasting symptomatic effects.^127^ A phase III, multicenter, RCT study (Exenatide‐PD3) adopted a “long‐term simple” design to evaluate the cumulative advantage secondary to prolonged treatment exposure (96 weeks).^128^ No significant differences in the progression of motor symptoms between treatment and placebo groups were detected, and no evidence to support any subgroup difference according to age, sex, age at diagnosis or motor stage.^168^

Safety and efficacy of NLY01, a brain‐penetrant, long‐lasting version of exenatide, were tested in early‐stage, drug naïve PD patients, with no improvement in motor and nonmotor features in the treated arm compared to placebo, although in this case a subgroup analysis suggested possible motor benefits in younger participants.^169^

Results from another phase II RCT showed that lixisenatide treatment was associated with milder progression of motor symptoms than placebo at 12 months in patients with early‐stage PD, but gastrointestinal side effects such as nausea and vomiting were reported in 46% and 13% of patients, respectively.^129^ Two additional phase II RCTs are currently underway and investigate the effects of liraglutide (ClinicalTrials.gov ID: NCT02953665) and semaglutide (GIPD; ClinicalTrials.gov ID: NCT03659682) in PD.

Overall, the contrasting results with the GLP‐1RA molecules tested so far do not enable definite conclusions. The results from the Exenatide‐PD3 trial are disappointing, but further analyses of biofluids from participants will shed light on the extent of target engagement and biological effects of the drug, and future post‐hoc analyses might identify subgroups of individuals according to biochemical features who benefitted more from exenatide than others.^168^ These data could help future researchers refine participants’ selection and outcomes’ choices in trials testing exenatide or other GLP‐1RA molecules.

## Conclusions

In the last 20 years, research into the gut‐brain axis has broadened our understanding of the pathogenesis, phenotypic heterogeneity and clinical trajectories of PD. Building upon the seminal Braak hypothesis, current models of PD pathogenesis support the existence of a body‐first subtype of PD where pathological changes might originate in the gut years or even decades prior to brain dysfunction. For this subset of individuals, alterations in the gut microbiome composition and dysfunction of EECs might contribute to the initial pathogenic alterations and potentially affect disease progression.

Research into the microbiome‐gut‐brain axis has paved the way for the identification of novel therapeutic targets, including symptomatic, disease‐modifying and prophylactic treatments, in PD. These treatments might be particularly effective in addressing troublesome, non‐motor symptoms such as constipation, but evidence from phase III RCTs, with appropriate study design and patient selection, is needed.

## Author Roles

(1) Research project: A. Conception, B. Organization, C. Execution; (2) Manuscript: A. Writing of the first draft, B. Review and Critique.

E.M.: 1A, 1B, 1C, 3A.

A.H.V.S.: 3B.

P.B.: 1A, 1C, 3B.

## Disclosures

**Ethical Compliance Statement:** Institutional review board was not necessary for his work. Informed patient consent was not necessary for this work. We confirm that we have read the Journal's position on issues involved in ethical publication and affirm that this work is consistent with those guidelines.

**Funding Sources and Conflicts of Interest:** This research was funded in part by Aligning Science Across Parkinson's (Grant number: ASAP‐000420) through the Michael J. Fox Foundation for Parkinson's Research (MJFF) and by the EU Joint Programme—Neurodegenerative Research (JPND) through the MRC grant code MR/T046007/1. For the purpose of open access, the author has applied a CC BY 4.0 public copyright license to all Author Accepted Manuscripts arising from this submission. The authors declare that there are no conflicts of interest relevant to this work.

**Financial Disclosures for the Previous 12 Months:** EM, PB have nothing to disclose. AHVS has provided paid consultancy to Capsida, Neurocrine and Auxilius, is the Chief Investigator of the ambroxol phase III study and a Principal Investigator of the MOVES‐PD study.

## Data Availability

Data sharing not applicable to this article as no datasets were generated or analysed during the current study.
